# Protocol for a multi-site randomized controlled trial of a stepped-care intervention for emergency department patients with panic-related anxiety

**DOI:** 10.1186/s12888-022-04387-z

**Published:** 2022-12-16

**Authors:** Sharon C. Sung, Leslie Lim, Swee Han Lim, Eric A. Finkelstein, Steven Lim Hoon Chin, Annitha Annathurai, Bibhas Chakraborty, Timothy J. Strauman, Mark H. Pollack, Marcus Eng Hock Ong

**Affiliations:** 1 Duke-NUS Medical School Singapore, 8 College Road, Singapore, 169857 Singapore; 2 Singapore General Hospital, Outram Road, Singapore, 169608 Singapore; 3 Changi General Hospital, 2 Simei Street 3, Singapore, 529889 Singapore; 4 Sengkang General Hospital, 110 Sengkang E Way, Singapore, 544886 Singapore; 5 National University of Singapore, 6 Science Drive 2, Singapore, 117546 Singapore; 6 Duke University, 2424 Erwin Road, Suite 1102, Durham, NC 27710 USA; 7 Duke University Medical Center, 10 Duke Medicine Cir, Durham, NC 27710 USA; 8 Rush University Medical Center, 1645 W. Jackson Blvd, Suite 400, Chicago, IL 60612 USA; 9 Sage Therapeutics, 215 First Street, Cambridge, MA 02142 USA

**Keywords:** Panic-related anxiety, Emergency department, Panic attack, Panic disorder, Chest pain, Stepped-care model, Cognitive behavioural therapy, Randomized controlled trial

## Abstract

**Background:**

Approximately 40% of Emergency Department (ED) patients with chest pain meet diagnostic criteria for panic-related anxiety, but only 1–2% are correctly diagnosed and appropriately managed in the ED. A stepped-care model, which focuses on providing evidence-based interventions in a resource-efficient manner, is the state-of-the art for treating panic disorder patients in medical settings such as primary care. Stepped-care has yet to be tested in the ED setting, which is the first point of contact with the healthcare system for most patients with panic symptoms.

**Methods:**

This multi-site randomized controlled trial (RCT) aims to evaluate the clinical, patient-centred, and economic effectiveness of a stepped-care intervention in a sample of 212 patients with panic-related anxiety presenting to the ED of Singapore’s largest public healthcare group. Participants will be randomly assigned to either: 1) an enhanced care arm consisting of a stepped-care intervention for panic-related anxiety; or 2) a control arm consisting of screening for panic attacks and panic disorder. Screening will be followed by baseline assessments and blocked randomization in a 1:1 ratio. Masked follow-up assessments will be conducted at 1, 3, 6, and 12 months. Clinical outcomes will be panic symptom severity and rates of panic disorder. Patient-centred outcomes will be health-related quality of life, daily functioning, psychiatric comorbidity, and health services utilization. Economic effectiveness outcomes will be the incremental cost-effectiveness ratio of the stepped-care intervention relative to screening alone.

**Discussion:**

This trial will examine the impact of early intervention for patients with panic-related anxiety in the ED setting. The results will be used to propose a clinically-meaningful and cost-effective model of care for ED patients with panic-related anxiety.

**Trial registration:**

ClinicalTrials.gov NCT03632356. Retrospectively registered 15 August 2018.

## Background

Panic disorder affects approximately 2% of the general population worldwide [1], and is associated with significant disability [2], high medical costs [3], and poor long-term outcomes [4]. The cardinal symptom of panic disorder is recurrent panic attacks consisting of symptoms such as chest pain, heart palpitations, shortness of breath, and other physical symptoms that may be interpreted by the sufferer as dangerous and indicative of a serious medical problem. Due to this misconception, patients with panic attacks often seek care in the emergency department (ED), rather than in psychiatric clinics or other mental health settings [5, 6]. A recent survey found that 34.5% of ED patients with non-cardiac chest pain met diagnostic criteria for panic attacks and 77% of these patients reported that they had visited the ED following a panic attack [7]. Despite the fact that up to 40% of ED patients with chest pain meet criteria for panic-related anxiety (i.e., panic attacks or panic disorder), only 1–2% of these patients are typically evaluated and treated for anxiety in this setting [6, 8]. Follow-up studies indicate that ED patients presenting with chest pain and panic attacks are at increased risk for developing panic disorder following discharge from the ED [9, 10]. Earlier identification and treatment of panic attacks when patients first visit the ED may help to prevent later progression to panic disorder, as well as poor long-term clinical outcomes and unnecessary recurrent ED visits for panic symptoms.

International clinical practice guidelines support the use of cognitive behavioural therapy (CBT) and antidepressant medications as first-line treatments for patients with panic disorder [11–15]. CBT for panic-related anxiety involves patient education regarding the nature and physiology of the panic response, techniques designed to alter catastrophic misinterpretations of panic symptoms, and gradual exposure to panic-related body sensations and avoided situations. Although pharmacotherapy and CBT show similar results in terms of short-term treatment efficacy, CBT has a more durable and sustained response following treatment discontinuation. It is also more cost-effective, has no serious adverse side effects, and fewer dropouts compared to pharmacotherapy [16, 17]. Furthermore, brief CBT protocols (5–7 sessions) have shown clinical and patient-centred outcomes that are equivalent to lengthier protocols [18].

A small number of studies have evaluated the impact of educational, pharmacological, and CBT interventions for patients with panic disorder initiated in the ED [19–21]. Although sample sizes were modest, the results have been promising with respect to clinical outcomes [19–21] and incremental cost-effectiveness [20, 22]. Meta-analytic data indicates that patients with panic symptoms have the best response to psychological interventions when implemented early in the course of illness [23]. However, only a few studies have tested the impact of early interventions on ED patients with panic attacks [24–26]. Participants who received psychoeducation and exposure instruction reported significant improvements with respect to depression symptoms and frequency of panic attacks [24], as well as decreased frequency of subsequent ED visits, and increased participation in psychiatric outpatient treatment [25, 26].

### Study rationale

**Fig. 1:**
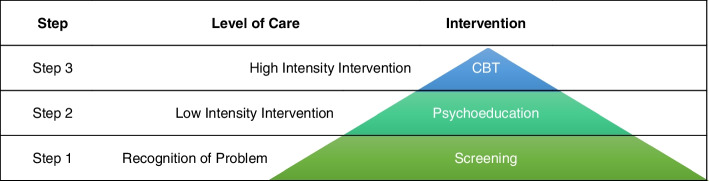
Recommended interventions at each level of a stepped-care model for treatment of patients with panic-related anxiety [14]

The current treatment gap for ED patients with panic-related anxiety constitutes a significant public health problem. It highlights the need to implement targeted early interventions that can improve clinical and patient-centred outcomes while reducing panic-related emergency medicine costs [27]. Tailored treatment via a ‘stepped-care’ model is now recommended as the state-of-the art for treating patients with panic-related anxiety [14] (see Fig. 1). In a stepped-care model, all patients start with a low-intensity evidence-based intervention. Progress is monitored and patients who do not respond adequately are subsequently ‘stepped up’ to a higher intensity treatment. Preliminary studies of stepped-care for panic disorder in primary care clinics have shown clinical effectiveness, patient-centred effectiveness, and cost-effectiveness relative to usual care [28, 29]. However, stepped-care for panic-related anxiety has yet to be tested in the ED setting, which is the first point of contact with the healthcare system for most patients with panic symptoms [6].

### Overall objectives

The main objectives of this study are to evaluate the clinical, patient-centred, and economic effectiveness of stepped-care for ED patients with panic-related anxiety, using a randomized controlled trial (RCT) design. The RCT will have two arms: 1) a treatment arm consisting of stepped-care intervention for panic-related anxiety (enhanced care pathway); and 2) a control arm consisting of screening for panic-related anxiety and discharge at the ED (usual care). In addition to the baseline assessment, the study will include follow-up visits at 1, 3, 6, and 12 months.

### Specific aims

*Aim 1 (Primary):* To evaluate the clinical effectiveness of a stepped-care intervention for ED patients with panic-related anxiety compared to screening alone.

*Outcomes under Aim 1:* The primary outcome under Aim 1 will be clinician-rated severity of panic symptoms, measured using the Panic Disorder Severity Scale (PDSS) [30] and the Clinical Global Impression Scale (CGI) [31]. Panic disorder will be diagnosed using the Structured Clinical Interview for DSM-5 (SCID) [32].

*Aim 2:* To evaluate the patient-centred effectiveness of a stepped-care intervention for ED patients with panic-related anxiety compared to screening alone.

*Outcomes under Aim 2:* We will examine health-related quality of life using the 12-item Short Form Health Survey (SF-12) [33], and the EuroQoL–5 Dimension (EQ-5D) [34]. We will assess psychiatric comorbidity using the Psychiatric Diagnostic Screening Questionnaire (PDSQ) [35], and healthcare utilization for panic-related symptoms (ED visits and hospital admissions) based on electronic medical record data in the 12 months following the intervention.

*Aim 3:* To evaluate the incremental cost-effectiveness of a stepped-care intervention for ED patients with panic-related anxiety compared to screening alone, from the health system perspective.

*Outcomes under Aim 3:* We will calculate the incremental cost-effectiveness ratio (ICER) of stepped-care intervention relative to screening alone, at 12 months from baseline. Direct healthcare costs will be calculated from electronic billing records and the effectiveness of the intervention will be assessed based on the conversion of SF-12 form to a quality of life weight using the Short Form Health Survey 6D (SF-6D) algorithm [36].

## Methods and analysis

### Study design

**Fig. 2:**
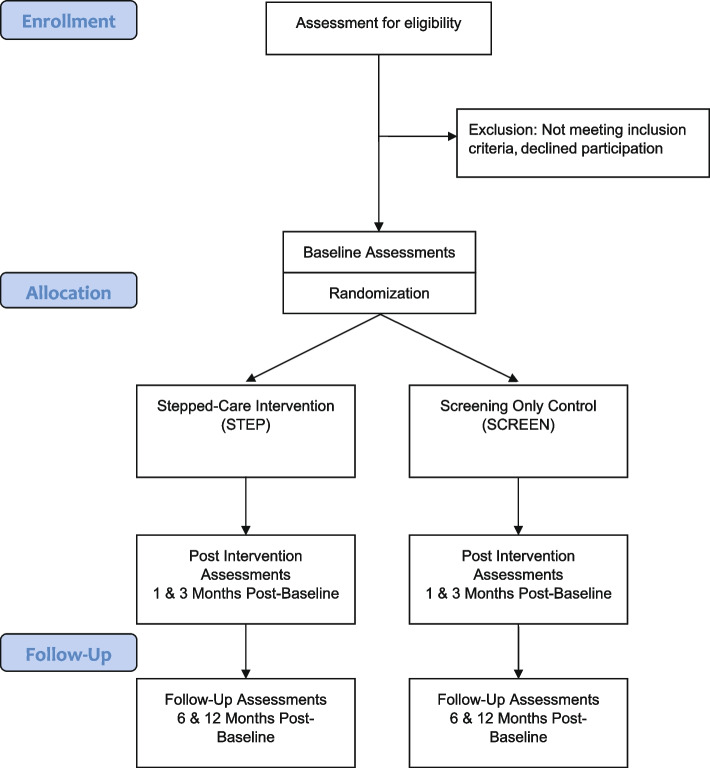
Study flow diagram

This 12-month superiority trial will follow a two-stage parallel group multi-site RCT design. Screening will be done at the first stage, followed by baseline assessments and block randomization in a 1:1 ratio to either the screening alone (SCREEN) or stepped-care (STEP) arms at the second stage. Only patients with a confirmed diagnosis of panic-related anxiety will be randomized at stage two. Masked follow-up assessments will be conducted by an independent evaluator at 1, 3, 6, and 12 months (See Fig. 2). We will follow the Consolidated Standards of Reporting Trials (CONSORT) guidelines for designing and reporting of randomized parallel-group non-pharmacologic intervention trials [37–39]. The trial protocol follows the SPIRIT recommendations and has been registered at clinicaltrials.gov (NCT03632356, 15 August 2018, current protocol version 4.0 dated 21 February 2019).

### Setting

The study will be conducted at multiple EDs in SingHealth, the largest public healthcare group in Singapore. Recruitment sites will include Singapore General Hospital (SGH), Changi General Hospital (CGH), and Sengkang General Hospital (SKH). All patients will be triaged based on severity using the Singapore Patient Acuity Category Scale [40]. Chest pain is the presenting complaint in up to 40% of triage level 1 and 2 cases (the most severe triage categories) [41].

### Participants & procedures

Participants will be recruited and assessed for eligibility by clinical research coordinators stationed in the ED during peak operating hours. To reduce the potential for attrition, participants will be given the option of completing follow-up assessments in person or by telephone.

Eligible participants at the first stage will be English- or Chinese-speaking men and women aged 21–70 years, who are assigned to triage level 2 (possibly critical) or 3 (minor emergency). They will need to present with at least one primary complaint consistent with a possible panic attack (e.g., tachycardia, chest pain, shortness of breath, dizziness, hyperventilation). Patients who are initially assigned to triage level 1 (critical illness requiring resuscitation) and subsequently downgraded to a non-life-threatening condition will also be eligible. We will exclude patients who are assigned to triage level 1 and deemed to have a life-threatening condition, who present with altered mental status (e.g., dementia, psychosis, substance intoxication or withdrawal) that would impact their ability to provide informed consent, who have symptoms with a clear cardiac or other medical cause (as confirmed by laboratory results and the ED physician), and who are unable or unwilling to complete the study procedures (See Table 1).

**Table 1:** Inclusion and exclusion criteria at each stage of the study

Stage 1: Screening	
*Inclusion Criteria:* 1. Male or female 2. 21+ years of age 3. Triage level 1 (not life-threatening condition), 2, or 3 4. English or Mandarin speaking 5. Able to provide informed consent and read study materials 6. Presenting complaint of chest pain, palpitations, dizziness, or breathing difficulties 7. Score ≥ 3 on the CDR screener	*Exclusion Criteria:* 1. Altered mental status (dementia, psychosis, substance intoxication/withdrawal) 2. Prisoner or police case 3. History of psychosis or schizophrenia or actively suicidal 4. Life-threatening condition requiring resuscitation 5. Not English- or Mandarin-speaking 6. Unwilling or unable to complete study procedures 7. Clear organic cause for panic symptoms as symptoms of potentially life threatening organic disease (e.g., acute coronary syndrome, STEMI, NSTEMI, angina pectoris), pneumothorax, aortic dissection, pulmonary embolism, tumour, effusion, rib fractures, pneumonia as determined by the ED physician
Stage 2: Randomization	
*Inclusion Criteria:* 1. Diagnosis of panic attack or panic disorder confirmed by the SCID interview 2. Willing to enter randomized trial	*Exclusion Criteria:* 1. Does not meet criteria for panic attack or panic disorder based on the SCID interview 2. Received CBT for panic symptoms in the last 12 months 3. Unwilling to enter randomized trial

In order to maximize generalizability to the broad group of patients with panic symptoms who present to the ED, we will not exclude patients who have received other types of psychotherapy for panic symptoms or those who are being treated with antidepressant medications. However, we will assess for these treatments at each time point and we will control for ancillary treatments in subsequent analyses. Patients who have received CBT for panic symptoms in the prior 12 months will be excluded.

### Interventions

Participants in both the SCREEN and STEP arms will follow the typical care pathway in the ED, which includes medical evaluation of their presenting complaint, any investigations ordered by the ED physician, reassurance that their symptoms are not indicative of acute coronary syndrome or other life-threatening illnesses, and outpatient follow-up as recommended at discharge. Participants in the SCREEN arm will be given the results of the screening interview, but no other protocol interventions. Participants in the STEP arm will be given a 1-hour psychoeducation session consisting of information regarding the nature and causes of panic attacks and behavioural strategies to manage future attacks. They will also be provided with written materials for future reference. Patients in the STEP arm who do not improve with psychoeducation will receive CBT based on a brief manualized protocol consisting of five weekly 1-hour individual sessions; the sessions will focus on cognitive restructuring and exposure to panic symptoms [42]. The psychoeducation and CBT sessions will be delivered by a trained clinical psychologist in either English or Mandarin, depending on the patient’s preference.

### Outcome measures

Outcome measures were selected to 1) provide adequate coverage of the core symptoms of panic attacks, panic disorder, and comorbid psychiatric conditions (e.g., depression, generalized anxiety disorder, substance use disorders), 2) assess disability and quality of life as well as symptom severity, and 3) have acceptable psychometric properties in English and Chinese. Whenever possible, we chose outcome measures that have been widely used in other clinical trials on panic disorder to facilitate comparison with other studies (i.e., in subsequent meta-analyses). In addition to clinician-rated and patient-reported outcome measures, we will collect service-use data from patient medical and billing records, and will ask patients to report on their use of ancillary treatments (e.g., other types of counselling, traditional Chinese medicine, stress management programs) and psychotropic medications at each assessment visit.

Clinician-rated outcomes: We will use the following clinician-rated assessment instruments to assess the outcomes under Aim 1.

*Clinical Decision Rule (CDR)*: The study team has developed a brief 7-item screening tool that can quickly distinguish between ED patients with or without panic-related anxiety [8]. The CDR consists of the following symptoms: 1) palpitations, pounding heart, or accelerated heart rate, 2) derealization, 3) paresthesia, 4) sensations of shortness of breath or smothering, 5) chills or hot flushes, 6) feeling dizzy, unsteady, lightheaded, or faint, and 7) fear of losing control or going crazy. The CDR is scored by assigning 1 point for each positive symptom. It has demonstrated excellent overall separation between patients with or without panic-related anxiety (area under the receiver operating characteristic curve = 0.90). At a cut- off score of 3 or greater, the CDR has a good balance between sensitivity (81.7%) and specificity (87.9%) [8].

*Panic Disorder Module of the Structured Clinical Interview for DSM-5 (SCID)*: The SCID is the gold standard tool for the reliable diagnosis of Axis I psychiatric disorders in clinical populations [32]. The study team has extensive experience using the panic disorder module of the SCID in previous ED studies [8, 43]. We found that inter-rater reliability was excellent for panic attacks (κ = 1.00) and panic disorder (κ = 0.82), indicating that the interviewers were able to assess panic symptoms with a high degree of accuracy.

*Panic Disorder Severity Scale (PDSS)*: The PDSS is a 7-item semi-structured interview to evaluate panic symptom severity [30]. It contains items that assess the frequency of panic attacks, level of distress during panic attacks, anticipatory anxiety, agoraphobic fear and avoidance, interoceptive fear and avoidance, impairment of occupational functioning, and impairment of social functioning. Each item is rated on a 0 (none/mild) to 4 (extreme/severe) scale. The English and Chinese versions of the PDSS have demonstrated good convergent/discriminant validity, internal consistency, inter-rater reliability, and test-retest reliability [30, 44–46]. PDSS scores are sensitive to treatment response and are recommended for treatment monitoring [12].

*Clinician Global Impression (CGI) severity scale*: The CGI is a clinician-rated instrument used to assess global severity of symptoms [31]. The CGI ranges from 1 (normal, not at all ill) to 7 (among the most extremely ill patients). The Singapore Clinical Practice Guidelines for Anxiety Disorders recommend using the CGI to measure illness severity and treatment progress during consultations for anxiety disorders [12]. Specific anchor points are used to delineate the domains of information to be assessed in scoring the CGI for patients with panic disorder [47]. The following parameters are assessed: number and frequency of panic attacks, intensity of anticipatory anxiety, degree of phobic avoidance, and functional impairment. The panic CGI has been successfully used in clinical trials for panic disorder and has established an inter-rater reliability of 0.89 [47].

Patient-reported outcomes: We will use the following instruments to assess the patient-centred outcomes under Aims 2 and 3. All patient-reported outcomes have strong psychometric properties, are available in English and Chinese, and have been used in local studies conducted by the study team.

*Short Form Health Survey (SF-12)*: The SF-12 is a reliable and valid 12-item self-report questionnaire that evaluates eight facets of health-related quality of life [33, 48]. It includes subscales to assess physical functioning, role limitations due to physical health problems, role limitations due to emotional problems, bodily pain, general health, vitality, social functioning, and mental health. The SF-12 will be used to assess health-related quality of life by converting the results into a quality of life weight using the SF-6D algorithm [36].

*Psychiatric Diagnostic Screening Questionnaire (PDSQ)*: The PDSQ is a reliable and valid self-report diagnostic questionnaire that has been widely used to assess the most common psychiatric disorders in outpatient settings, including major depressive disorder, generalized anxiety disorder, panic disorder, posttraumatic stress disorder, alcohol abuse/dependence, drug abuse/dependence, psychosis, bulimia/binge eating disorder, and somatization disorder [35]. It includes 125 yes/no items that can be summed for a total score, which functions as a global indicator of psychopathology.

*EuroQoL- 5 Dimension (EQ-5D)*: The EQ-5D is a patient self-report instrument that evaluates health-related quality of life [34] . It includes one question each to assess mobility, self-care, usual activities, pain/discomfort, and anxiety/depression, as well as a Visual Analog Scale (VAS) that asks respondents to rate their perceived health status from 0 (the worst possible health status) to 100 (the best possible health status).

### Criteria for ‘stepping up’ in the stepped-care intervention arm

The decision to step-up interventions for patients in the STEP arm will be guided by remission status as assessed by the PDSS and CGI. Acute remission status is defined by a CGI score of 1 or 2 (no disorder or borderline disorder) and no panic attacks for a one-week period. Full remission is defined by maintenance of this status for two consecutive months [49]. Participants who show acute remission of panic symptoms one month after completing the psychoeducation session will enter the follow-up phase. Those who do not show acute remission of panic symptoms at one-month post-psychoeducation or return to the ED with panic symptoms within three months will be stepped up to receive the 5-session CBT protocol.

### Treatment fidelity

All CBT sessions in the STEP arm will be recorded with patient permission. A subset of 20% of the recordings will be reviewed and scored by for adherence to the study protocol using the CBT subscale of the Collaborative Study Psychotherapy Rating Scale (CSPRS), a psychometrically-sound tool for assessing adherence to CBT intervention in clinical trials [50].

### Participant timeline

Participants in both arms will be involved in the trial for a period of 12 months. Following recruitment at baseline at the ED, masked follow-up assessments will be conducted by an independent interviewer at 1, 3, 6, and 12 months. The timeline of study assessments is shown in Table 2. Participants in the STEP arm will receive psychoeducation within 1 week of discharge from the ED and those who are stepped-up will receive 5 weekly sessions of CBT between the 1-month and 3-month follow-up.

**Table 2:** Timeline of study assessments

Construct	Outcome Measure	Time point					
		Screening	Baseline	1 month	3 months	6 months	12 months
Panic Screening	CDR^a^	x					
Panic Attack/Disorder Diagnosis	SCID Panic Module^a^		x	x	x	x	x
Panic Symptom Severity	PDSS^a^		x	x	x	x	x
	CGI^a^		x	x	x	x	x
Psychiatric Comorbidity	PDSQ^b^		x	x	x	x	x
Health-Related Quality of Life	SF-12^b^		x	x	x	x	x
Healthcare Utilization	Electronic medical record						x
Direct Healthcare Costs	Electronic billing data						x
Cost Effectiveness	SF-6D/EQ-5D^b^		x	x	x	x	x

^a^Clinician-rated outcomes. ^b^Patient-reported outcomes

*CDR* Clinical Decision Rule; *SCID5 RV* Structured Clinical Interview for DSM-5; *PDSS* Panic Disorder Severity Scale; *CGI* Clinical Global Impressions Severity Scale; *PDSQ* Psychiatric Diagnostic Screening Questionnaire; *SF-12* Short Form Health Survey; *EQ-5D* EuroQoL- 5 Dimension

### Sample size

We will aim to recruit a randomized sample of 212 participants. Sample size was estimated based on change in panic symptom severity with PDSS score at 1-month post-intervention as the primary outcome. Previous studies of the 5-session CBT protocol for patients with panic disorder showed relatively large effect size changes on the PDSS from pre- to post-intervention [42] (Cohen’s d = 2.03–2.62). Based on previous literature, we expect a more modest effect size change for the psychoeducation intervention alone [24]. Assuming the true difference between treatments to be 0.516 times the common standard deviation (i.e., Cohen’s d = 0.516, which is approximately Cohen’s medium effect size), a final sample size of 80 participants in each group (after accounting for 25% attrition) would ensure 90% power at a two-sided 0.05 significance level.

### Randomization

Patients will be randomly allocated to one of the two arms based on a code generated using the open-source software R (https://www.r-project.org) and contained within sequential sealed envelopes. We will stratify the participants by the diagnosis of panic attack or panic disorder. Within each stratum, we will apply a permuted block randomization scheme with a block size of 4 and a 1:1 randomization ratio between the screening alone (SCREEN) or stepped-care intervention (STEP) arms. This permuted block randomization will ensure balance in the trial. The randomization block will be generated before commencing the study at the various sites. The study coordinator will proceed to open the randomisation envelopes to reveal the allocation only after a patient has been enrolled in the trial.

### Masked follow-ups

Masking will be carried out by the individual study site coordinators. The follow up assessments will be conducted by a masked team member who is blind to allocation. However, as this is a psychotherapy trial, it will not be possible to blind participants with respect to their own allocation.

### Data management

Study coordinators will enter data collected into a password-protected database developed for ease of entry and reduction of error. All data will be error-checked and transferred to a statistical modelling software by the biostatistician in the team. Study data will be kept confidential and de-identified, and only authorized study staff will have access.

### Data analysis

We will use the following statistical methods to evaluate the specific aims.

*Aim 1: To evaluate the clinical effectiveness of stepped-care* versus *screening alone:* All analyses will be based on the intention-to-treat (ITT) principle. We will employ a mixed model approach to analyse the primary outcome variable, with repeated measurements taken at the 1-, 3-, 6- and 12-month follow-up visits. These models allow for different number of observations per subject, use all available data on each subject, and are unaffected by randomly missing data. We will examine the fixed categorical effects of treatment pathway (STEP, SCREEN), visit time (1, 3, 6, and 12 months), and treatment pathway-by-visit time interaction. The restricted maximum likelihood approach will be utilized for estimation. Significance tests will be based on least squares means using a two-sided alpha = 0.05. Primary treatment comparisons will be contrasts in least squares means between treatment groups at the endpoint visit. The CGI scores and SCID diagnoses will be analysed in a similar fashion. As a follow-up to the ITT analyses, we will also conduct a completer analysis. Participants in the STEP arm who are stepped up to CBT and complete four out of five of the CBT sessions will be deemed completers. Those in the STEP arm who complete the psychoeducation session and are not stepped-up to CBT will also be considered completers for the purpose of analysis.

*Aim 2: To evaluate the patient-centred effectiveness of stepped-care* versus *screening alone:* We will use the same mixed model approach as outlined under Aim 1 to analyse data from the patient-reported outcomes (SF-12, EQ-5D, and PDSQ).

*Aim 3: To assess the cost-effectiveness of stepped-care* versus *screening alone from the health system perspective:* We will estimate the ICER for the STEP arm participants relative to the SCREEN arm participants at 12 months from baseline. The ICER is calculated as the difference between the costs of the two strategies divided by the difference in their effectiveness. Costs from the health system perspective will be the difference in average costs between those in the STEP and SCREEN arms, based on hospital billing data. The difference in effectiveness will be based on the quality of life weights generated by applying the SF-6D algorithm to scores from the SF-12 survey. The STEP intervention will be considered dominant over SCREEN if it incurs less costs and generates higher quality-adjusted life years (QALYs). STEP will be considered cost effective relative to SCREEN if the ICER is below the cost-effectiveness threshold. We will adopt the National Institute for Health and Care Excellence’s cost-effectiveness threshold of £20,000–30,000 per QALY gained, which roughly translates into $63,313 SGD per QALY gained [51].

### Data monitoring

The Principal Investigator and Co-Investigators will be responsible for data and safety monitoring. The study coordinators will meet with the Principal Investigator fortnightly to provide recruitment updates and to review any adverse events or serious adverse events that occur during the study period. Due to the nature of the study procedures (i.e., screening interviews, questionnaire assessments, CBT interventions), there is not a high risk of adverse events or serious adverse events. In the case of such events, the Principal Investigator will be responsible for reporting any adverse events to the relevant authorities, regulatory bodies, and institutions as required.

## Discussion

The present study will evaluate the clinical, patient-centred, and economic effectiveness of a stepped-care intervention for ED patients with panic-related anxiety. We are aware of no prior studies seeking to evaluate screening and stepped-care for this patient population. In the short term, we expect that expedient care of patients presenting to the ED with panic attack symptoms will lead to improved clinical outcomes (i.e., symptom remission, improved quality of life) as well as reduced burden on the healthcare system due to reductions in unnecessary recurrent ED visits, investigations, and hospital admissions. In the long term, early identification and treatment of patients with panic-related anxiety may help to decrease waiting times at the ED and increase the availability of beds for patients needing urgent care. For example, a recent study from Taiwan found that psychiatric treatment of patients with panic disorder translated into a 30% reduction in related ED costs [27]. The two strategies being evaluated in the proposed RCT have the potential to significantly reduce healthcare costs while simultaneously improving patient outcomes.

## Study status

This trial commenced in June 2018. Baseline recruitment is still ongoing, with a planned end date of February 2023. Follow-up data collection will continue until February 2024.

## Data Availability

Not applicable.

## Abbreviations

*CBT*: Cognitive Behavioural Therapy
*CDR*: Clinical Decision Rule
*CGH*: Changi General Hospital
*CGI*: Clinical Global Impression Scale
*CONSORT*: Consolidated Standards of Reporting Trials
*CSPRS*: Collaborative Study Psychotherapy Rating Scale
*ED*: Emergency Department
*EQ-*5D: EuroQoL-5 Dimension
*ICER*: Incremental Cost-effectiveness Ratio
*ITT*: Intention-to-treat
*PDSS*: Panic Disorder Severity Scale
*PDSQ*: Psychiatric Diagnostic Screening Questionnaire
*QALYs*: Quality-adjusted Life Years
*RCT*: Randomized Controlled Trial
*SCID*: Structured Clinical Interview for DSM-5
*SF-*6D: Short Form Health Survey 6D
*SF-*12: 12-item Short Form Health Survey
*SGH*: Singapore General Hospital
*SKH*: Sengkang General Hospital
*VAS*: Visual Analog Scale
